# A Unique Reverse Adaptation Mechanism Assists Bordetella pertussis in Resistance to Both Scarcity and Toxicity of Manganese

**DOI:** 10.1128/mBio.01902-21

**Published:** 2021-10-26

**Authors:** Jan Čapek, Ilona Procházková, Tomáš Matoušek, David Hot, Branislav Večerek

**Affiliations:** a Laboratory of Post-transcriptional Control of Gene Expression, Institute of Microbiology of the Czech Academy of Sciences, Prague, Czech Republic; b Institute of Analytical Chemistry of the Czech Academy of Sciences, Brno, Czech Republic; c CHU Lille, Institut Pasteur de Lille, Inserm, CNRS, UMR2014—US41—PLBS Plateformes Lilloises de Biologie & Santé, Université de Lille, Lille, France; Department of Veterinary Medicine

**Keywords:** *Bordetella*, pathogen adaptation, genome decay, manganese, oxidative stress, *Bordetella pertussis*

## Abstract

The ability of bacterial pathogens to acquire essential micronutrients is critical for their survival in the host environment. Manganese plays a complex role in the virulence of a variety of pathogens due to its function as an antioxidant and enzymatic cofactor. Therefore, host cells deprive pathogens of manganese to prevent or attenuate infection. Here, we show that evolution of the human-restricted pathogen Bordetella pertussis has selected for an inhibitory duplication within a manganese exporter of the calcium:cation antiporter superfamily. Intriguingly, upon exposure to toxic levels of manganese, the nonfunctional exporter becomes operative in resister cells due to a unique reverse adaptation mechanism. However, compared with wild-type (wt) cells, the resisters carrying a functional copy of the exporter displayed strongly reduced intracellular levels of manganese and impaired growth under oxidative stress. Apparently, inactivation of the manganese exporter and the resulting accumulation of manganese in the cytosol benefited the pathogen by improving its survival under stress conditions. The inhibitory duplication within the exporter gene is highly conserved among B. pertussis strains, absent from all other *Bordetella* species and from a vast majority of organisms across all kingdoms of life. Therefore, we conclude that inactivation of the exporter gene represents an exceptional example of a flexible genome decay strategy employed by a human pathogen to adapt to its exclusive host.

## INTRODUCTION

Upon infection, bacterial pathogens enter hostile and variable environments containing limited amounts of essential nutrients and, consequently, face several stresses. One of the major challenges is to sustain metal homeostasis and adapt to nutritional and oxidative stresses. Thus, as an important part of the response to infection, host organisms employ several mechanisms and strategies to unbalance the pathogen metabolism and metal homeostasis (1). Given the transition metal essentiality on one side and their metal-linked toxicity on the other, the host cells exploit in principle two opposite antimicrobial strategies. Either they intoxicate the cells by pumping metal into the pathogen milieu by dedicated transporters (2) or, as a part of so-called nutritional immunity, they deprive the pathogen of the essential metal by means of metal-sequestering proteins (3, 4) and efflux pumps that transport metals out of the bacterium-containing phagosomes.

Manganese (Mn) is an important transition metal and essential micronutrient for bacterial pathogens (5). It is an efficient catalyst in redox transactions and a cofactor in various enzymes (for a review, see references 6 and 7). Importantly, there is growing evidence that bacterial pathogens use Mn as an important element in the defense against oxidative stress (8, 9). Indeed, mutants defective in Mn import displayed increased sensitivity to reactive oxygen species (10, 11). Therefore, to prevent or attenuate the infection, host cells deprive pathogens of extracellular Mn through different mechanisms. Neutrophils and macrophages produce calprotectin, an S100 protein family member consisting of S100A8 and S100A9 subunits (12), which efficiently scavenges Mn and zinc from the host environment and sensitize the pathogen toward oxidative stress effects (13). Furthermore, phagocytic cells can withhold the Mn from internalized bacteria by another mechanism based on the divalent metal exporter NRAMP1 (14, 15). While the procedures of Mn deprivation are well characterized, the molecular mechanisms of host-mediated intoxication of invading cells are poorly understood. Mn exporters have been discovered recently in several pathogenic bacteria, and mutants lacking these exporters displayed attenuated virulence and reduced ability to survive within the host environments (16–19). However, it remains to be clarified whether these effects resulted from Mn intoxication.

Given the role of Mn in redox and metal homeostasis, its transport is tightly regulated (5). Import of Mn into the bacterial cell is facilitated by bacterial homologues of the NRAMP1 transporter family (e.g., MntH [20]) or by ATP-binding cassette (ABC)-type transporters (e.g., MntABC [21]), while the export is accomplished by various efflux pumps belonging to several protein superfamilies, such as lysine exporters (e.g., MntP), cation diffusion facilitators (e.g., MntE), and P-type ATPases (e.g., CtpC) (16, 22–24). Mn trafficking is controlled by the cognate transcriptional regulators MntR (21, 25, 26) and Mur (27, 28) as well as by global regulators involved in metal homeostasis and oxidative stress response, such as Fur and OxyR (23, 29, 30). Furthermore, regulatory RNAs such as riboswitches have been shown to play essential roles in balancing the Mn homeostasis. Riboswitches are highly structured *cis*-acting groups of noncoding RNAs positioned within the 5′-untranslated regions of, and are cotranscribed with, the genes they regulate (31). Among other metabolites, riboswitch aptamers also recognize and bind metals and, consequently, riboswitches were found to control expression of metal transporters and to contribute to regulation of metal homeostasis (32). Recently, an Mn-responsive riboswitch was shown to control expression of an Mn exporter in Escherichia coli (33) and Lactococcus lactis (34).

Bordetella pertussis is a strictly human reemerging pathogen and causative agent of whooping cough (35). B. pertussis has evolved from a B. bronchiseptica-like ancestor (36), and as a part of the ongoing adaptation to its host, the B. pertussis genome was continuously rearranged, reduced, and decayed (37–39). Thus, the B. pertussis genome contains hundreds of pseudogenes, i.e., nonfunctional genes inactivated mostly by an insertion sequence element transposition and inversion or by deletion/insertion of one or multiple nucleotides (37). Our analysis of the primary transcriptome of B. pertussis revealed hundreds of noncoding transcripts, including several riboswitches (40). Here, we describe an Mn-sensing riboswitch that controls the expression of the downstream *BP3410* gene encoding a nonfunctional Mn exporter. Intriguingly, although the functionality of the exporter gene was fully recovered in resister cells formed in the presence of toxic concentrations of Mn, the wild-type (wt) strain carrying the decayed copy of the exporter displayed improved growth under oxidative stress compared to resister cells. We propose that during infection, when access to Mn is limited, the nonfunctional Mn exporter is beneficial to B. pertussis survival.

## RESULTS

### Expression of the *BP3410* gene is controlled by an Mn^2+^-responsive riboswitch.

**FIG 1:**
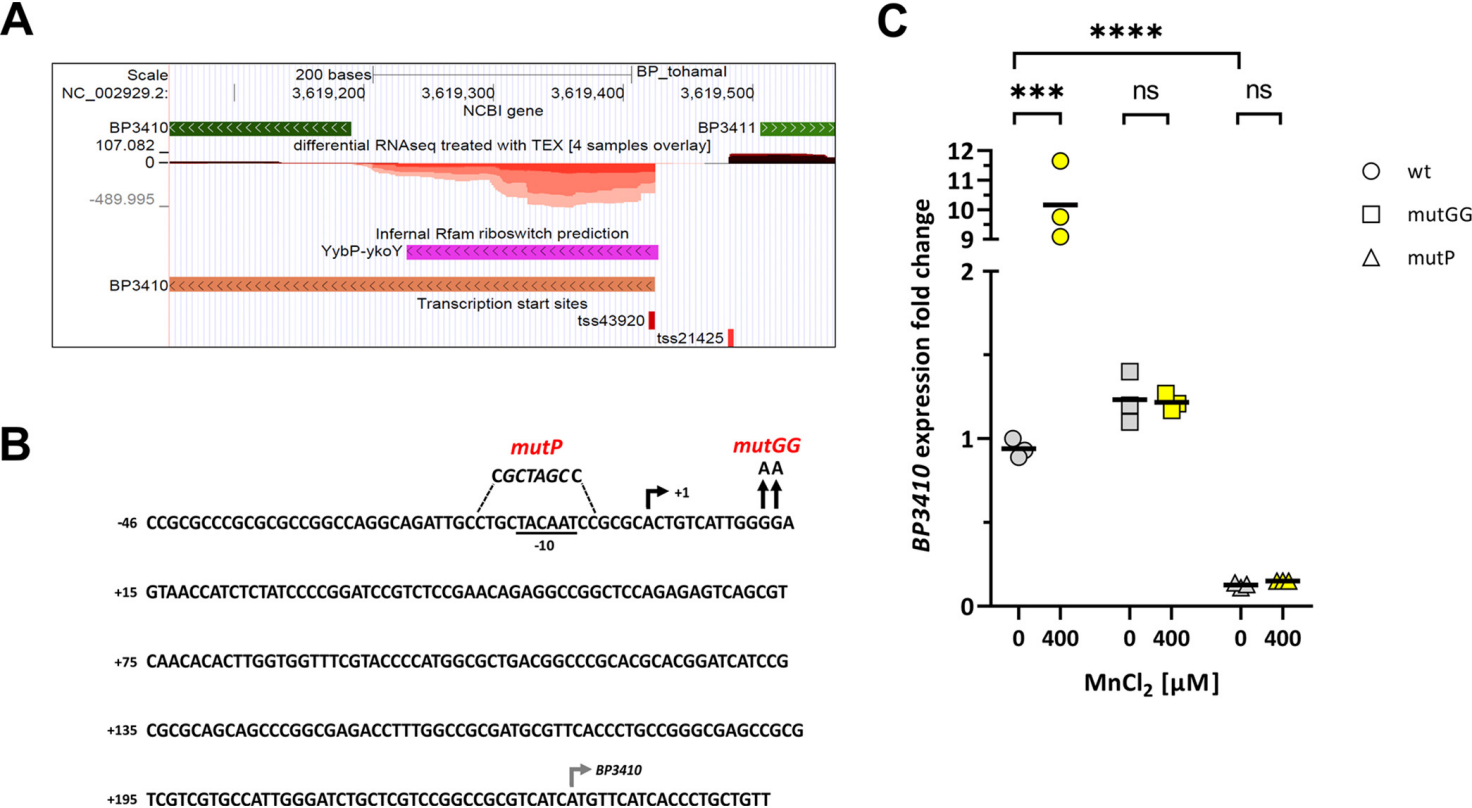
Mn-responsive riboswitch *yybP*-*ykoY* affects the expression of *BP3140* gene. (A) A detailed view of the genome browser (http://bit.ly/2Euo5HZ) (40) showing the dRNA-seq normalized data set of the region between genes *BP3410* and *BP3411* (green arrows). The plot shows the sequencing depth of expression of the positive-strand (depth above the zero line, dark red) and the negative-strand (depth below the zero line, light red) expression, with different color intensities representing the different library replicates. The transcription start sites of the *BP3410* (TSS43920) and *BP3411* (TSS21425) genes and the predicted riboswitch region (purple arrows) are also shown. (B) Nucleotide sequence map of the region upstream of the *BP3410* gene encompassing the riboswitch region. The transcription start site identified by dRNA-seq analysis (black rectangular arrow, +1), the plausible −10 promoter sequence (underlined), and the start codon of the *BP3410* gene (gray rectangular arrow) are shown. The mutations introduced into the riboswitch are indicated above the sequence. To construct the *BP3410_mutP_* strain, the sequence around the −10 site was altered to prevent transcription of the *BP3410* gene, and the introduced NheI restriction site is shown in italics. The *BP3410_mutGG_* mutant was created by replacing two adjacent G residues with A residues (depicted by vertical arrows). (C) RT-qPCR analysis was performed with total RNA isolated from three biological replicates of wt, *BP3410_mutP_*, and *BP3410_mutGG_* cells induced with 400 μM Mn (yellow symbols) for 60 min or left untreated (gray symbols, controls). The dot plot shows individual data points for all strains; the horizontal black lines represent mean values. Statistical analysis was performed using an unpaired two-tailed *t* test; ns, *P* > 0.05; ***, *P* < 0.0005; ****, *P* < 0.00005. Expression of *BP3410* in the first biological replicate of the wt strain grown in the absence of Mn was arbitrarily set to 1, and all other values are expressed relative to this value.

Our recent analysis of the primary transcriptome of Bordetella pertussis revealed a large variety of noncoding transcripts, including several riboswitch structures (40). One of the riboswitches was identified in the 5′-untranslated region (UTR) of the *BP3410* gene, which was annotated as a calcium:cation antiporter (Fig. 1A). Curiously, the riboswitch region overlapped a previously identified small RNA, BprK, that initially had been predicted by several bioinformatics tools and then confirmed by Northern blotting to be an approximately 200-nucleotide (nt) transcript (41). However, a search against the Rfam database confirmed that BprK constitutes an orphan *yybP-ykoY* riboswitch (E value of 8.5 × 10^−15^). These riboswitches are frequently found upstream of genes encoding transmembrane proteins involved in the transport of metals, and some of them are Mn responsive (33, 34). The Stainer-Scholte (SS) medium, used in our experiments to grow B. pertussis, is not supplemented with Mn (42), and therefore the presumed sRNA most likely represents a *BP3410*-specific transcript that terminates prematurely within the riboswitch structure in the absence of Mn. In support of this, our primary transcriptome analysis showed that the *BP3410* gene is transcribed exclusively from a single promoter upstream of the riboswitch and that this transcript originates from the negative strand as the A residue at position 3,619,424 (TSS43920 in Fig. 1A) within the B. pertussis genome (37, 40). At an appropriate distance from the transcriptional start site, we identified a plausible −10 promoter sequence (TACAAT) (Fig. 1B). Collectively, these data revealed that the *BP3410* gene is transcribed from the promoter upstream of the riboswitch; however, in the absence of Mn, the RNA polymerase stops within the riboswitch region.

To test this assumption experimentally, we analyzed the link between the riboswitch, Mn, and *BP3410* expression by quantitative PCR (qPCR) and constructed two mutants for this purpose. First, we mutated the plausible −10 region upstream of the riboswitch (*BP3410_mutP_* strain), and second, we mutated two conserved G residues within the riboswitch aptamer (*BP3410_mutGG_* strain) that were documented to be critical for Mn binding (33, 34) (Fig. 1B). Overnight cultures of wt, *BP3410_mutP_*, and *BP3410_mutGG_* strains were divided and further incubated either in the absence (controls) or presence of 400 μM Mn for 60 min. *BP3410* expression was then examined by reverse transcription-qPCR (RT-qPCR). As demonstrated in Fig. 1C, the addition of Mn induced the expression of the *BP3410* gene in the wt strain, while this effect was absent from both mutants. Notably, under noninducing conditions, *BP3410* expression was significantly reduced in the *BP3410_mutP_* strain compared with the wt and *BP3410_mutGG_* strains. This observation suggested that the inhibitory structure of the riboswitch does not completely disrupt transcription driven by the *BP3410* promoter even in the absence of Mn. It should be noted that the addition of different concentrations of calcium had no effect on *BP3410* gene expression (data not shown). Collectively, our results proved that the *BP3410* gene is transcribed from a single promoter upstream of the riboswitch and that binding of Mn to the riboswitch is required for induction of *BP3410* expression.

### The wild-type strain forms survivor colonies in the presence of toxic manganese concentrations.

**FIG 2:**
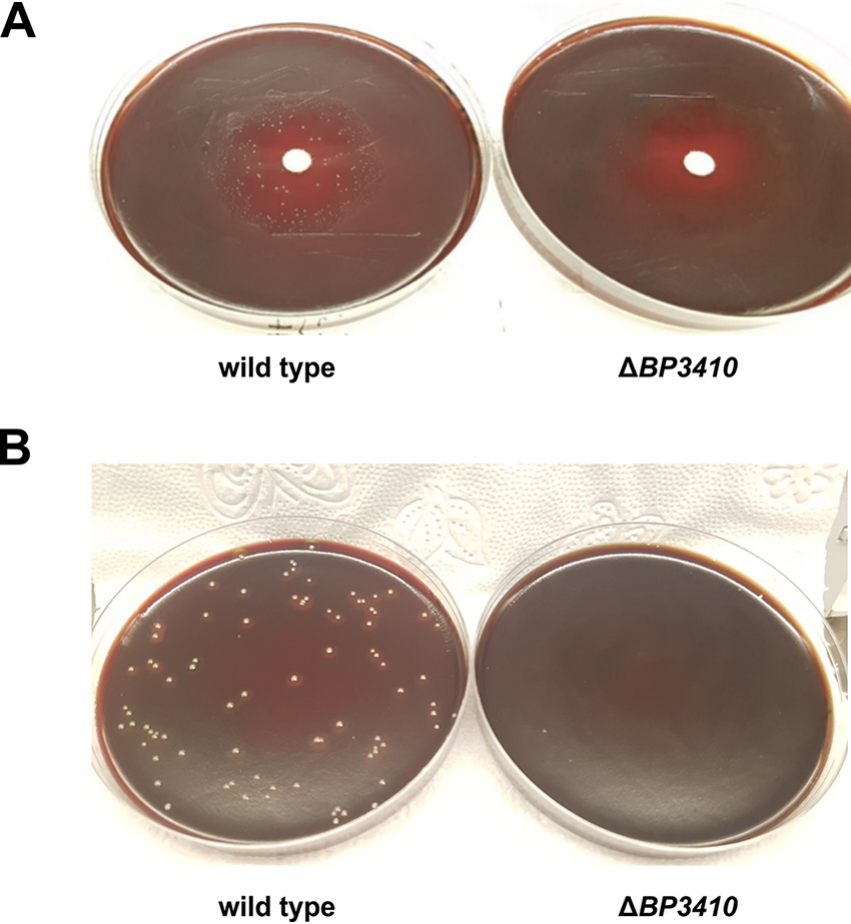
Growth of B. pertussis in the presence of toxic concentrations of manganese. (A) B. pertussis wt and Δ*BP3410* cells were grown in SS medium to late exponential phase, and then 100 μl of cell suspension (OD_600_ of suspension was set to 2) was plated on BG agar. Sensitivity to Mn was tested with a disc diffusion assay in which 10 μl of 100 mM MnCl_2_ was pipetted onto the disc. (B) B. pertussis wt and Δ*BP3410* cells were grown in SS medium to an OD_600_ of ≈2.0, and then 100 μl of each culture was plated on BG agar supplemented with 1 mM MnCl_2_.

The inducibility of *BP3410* expression by Mn, as well as the high similarity between the 5′-UTR of the *BP3410* gene and *yybP-ykoY* riboswitch, suggested that the BP3410 protein constitutes an Mn exporter despite its annotated function. To experimentally confirm this hypothesis, we constructed a markerless in-frame Δ*BP3410* deletion mutant of B. pertussis with the rationale that the mutant strain should exhibit a growth defect in the presence of toxic Mn concentrations. We used a disc diffusion assay to compare the sensitivity of the wt and Δ*BP3410* strains toward the gradient of Mn concentrations. Surprisingly, both strains exhibited similarly sized zones of inhibition around discs soaked with 10 μl of 100 mM Mn. However, in contrast to the plates inoculated with the Δ*BP341*0 strain, the plates inoculated with the wt strain revealed distinct colonies within the zone of inhibition (Fig. 2A). To corroborate this observation, we plated both wt and Δ*BP3410* cells on agar plates supplemented with 1 mM Mn. While we did not obtain any colonies of the mutant, plating out the wt strain yielded dozens of colonies (Fig. 2B) and allowed us to estimate that these survivors were formed at an approximate frequency of 1.06 × 10^−6^ ± 0.62 × 10^−6^.

### The Mn resisters lack the unique duplication within the *BP3410* gene that is highly conserved in B. pertussis.

**FIG 3:**
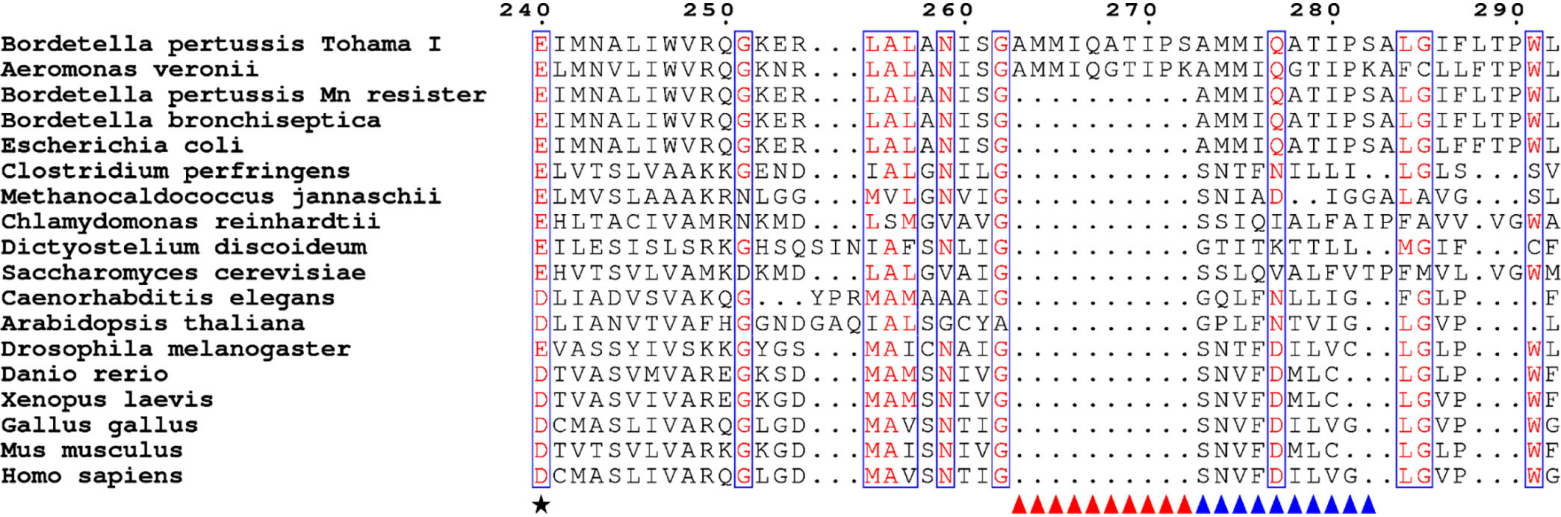
Conservation of duplication within the BP3410 exporter in other organisms. The amino acid sequences of the BP3410 exporter of B. pertussis and its selected homologues found in different organisms were aligned with the MUSCLE algorithm built in the MEGA software using the neighbor-joining clustering method. The black asterisk indicates a highly conserved negatively charged residue essential for cation transport. Aligned residues that can be considered similar based on their physicochemical distance at a given position are shown in red. Similar residues found in at least 70 % of the aligned sequences are enclosed in blue frames. The triangles mark the duplicated amino acid sequence present only in B. pertussis and three recent isolates of *A. veronii* (red triangles) and the sequence present in all homologous proteins (blue triangles). The amino acid residues are numbered according to the B. pertussis BP3410 protein. The complete alignment is presented in Fig. S1.

Our results indicated that B. pertussis cells can adapt to toxic concentrations of Mn. To clarify the genetic background of this adaptation, the *BP3410* locus was sequenced in several survivor cells and compared with the wt strain. Interestingly, all the survivors carried a 30-nt deletion within the coding region of the *BP3410* gene, resulting in a deletion of 10 amino acids. Closer inspection of this region revealed that this region is duplicated in the wt strain, whereas all survivors, here referred to as Mn resisters, contained only one copy of this 30-nt segment (Fig. 3). Furthermore, a comparison of the corresponding region in all fully sequenced B. pertussis strains deposited in GenBank showed that this duplication is present in almost 98 % of the strains. The calcium:cation antiporters represent a ubiquitous protein family found across all kingdoms of life. Nevertheless, as shown in Fig. 3, the observed duplication is highly specific only to B. pertussis, with the exception of three recent isolates of Aeromonas veronii (43).

10.1128/mBio.01902-21.1 FIG S1 Complete alignment of BP3410 exporter homologues in other organisms. The amino acid sequences of the BP3410 exporter of B. pertussis and its selected homologues found in different organisms were aligned with the MUSCLE algorithm built in the MEGA software using the neighbor joining clustering method. The black asterisk indicates a highly conserved negatively charged residue essential for cation transport. Aligned residues that can be considered similar based on their physicochemical distance at a given position are shown in red. Similar residues found in at least 70 % of the aligned sequences are enclosed in blue frames. The triangles mark the duplicated amino acid sequence present only in B. pertussis and three recent isolates of *A. veronii* (red triangles), and the sequence present in all homologous proteins (blue triangles). The amino acid residues are numbered according to the B. pertussis BP3410 protein. Download FIG S1, PDF file, 0.03 MB. Copyright © 2021 Čapek et al. 2021 Čapek et al. https://creativecommons.org/licenses/by/4.0/ This content is distributed under the terms of the Creative Commons Attribution 4.0 International license.

### Duplication in BP3410 is lost in cells growing in the presence of toxic Mn concentrations.

**FIG 4:**
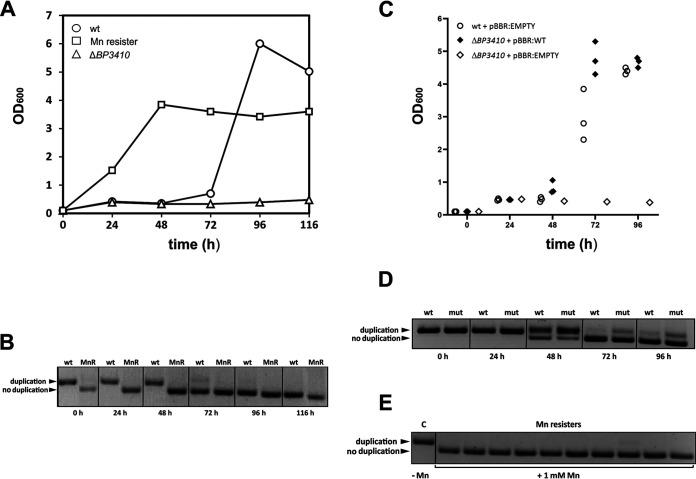
B. pertussis forms resister cells during growth in the presence of toxic concentrations of manganese. (A) B. pertussis wt, Δ*BP3410*, and Mn resister cells were grown in SS medium in the presence of 200 μM Mn. Optical density (OD_600_) was measured every 24 h, and samples were collected in parallel for PCR analysis. (B) Genomic DNA was isolated from wt and Mn resister (MnR) cells harvested in parallel with optical density measurements and used as a template for amplification of the region encompassing the duplication within the *BP3410* gene. PCR products that either contain or do not contain the duplication are indicated by arrowheads. (C) B. pertussis wt cells carrying an empty plasmid (white circles) and complemented Δ*BP3410* cells carrying a copy of the *BP3410* gene on the plasmid (black diamonds) were grown in triplicate cultures in SS medium supplemented with 200 μM Mn. As a control, a single culture of Δ*BP3410* cells carrying empty plasmid (white diamonds) was grown under the same conditions. The plot shows individual optical density values measured every 24 h. (D) Genomic DNA was isolated from the wt strain carrying an empty plasmid (wt) and the Δ*BP3410* mutant carrying plasmid-borne *BP3410* gene (mut) harvested in parallel with the optical density measurements (C) and used as a template for amplification of the region encompassing the duplication within the *BP3410* gene. PCR products that either contain or do not contain the duplication are indicated by arrowheads. (E) Genomic DNA was isolated from Δ*BP3410* cells carrying plasmid-borne *BP3410* gene harvested from plates without (control, C) or with 1 mM Mn (Mn resisters). PCR products either containing the duplication or not are indicated by arrowheads. The experiment was performed in three biological replicates. In each experiment, 10 resister clones were analyzed. A representative experiment is shown.

To gain better insight into the dynamics of duplication loss, we monitored the growth characteristics of the wt, Δ*BP3410*, and Mn resister strains in SS medium supplemented with 200 μM Mn. In parallel with the optical density measurements, we collected samples for isolation of genomic DNA and subsequent amplification of the region around the duplication by PCR. The Δ*BP3410* mutant reached a very low OD_600_ (≈0.3) and then ceased growth, whereas the Mn resister exhibited a standard growth curve (Fig. 4A). Interestingly, the wt strain grew similarly to the Δ*BP3410* mutant for the first 3 days, but after this rather long lag time of 72 h, it resumed growth and reached optical densities comparable to those of the Mn resister. PCR analysis of samples collected during incubation clearly showed that by the third day of cultivation, the wt cells carrying the 30-bp duplication were eliminated and replaced by the Mn resisters without the duplication (Fig. 4B). Apparently, growth of B. pertussis in the presence of toxic amounts of Mn did not resume until the duplication in the *BP3410* gene was lost. Nevertheless, to rule out the possibility that an additional mutation(s) was responsible for improved growth of the Mn resister, site-directed mutagenesis was used to construct a mutant strain lacking duplication in the *BP3410* gene. Compared with the spontaneously evolved resister, this mutant, here referred to as the constructed Mn resister (Mn resister_C_), displayed a nearly identical phenotype in the presence of toxic amounts of Mn (data not shown). To further corroborate our results, we tested whether ectopic expression of the *BP3410* gene would remedy the growth defect of the Δ*BP3410* strain observed in the presence of an inhibitory Mn concentration. Therefore, the Δ*BP3410* strain was complemented with the plasmid carrying the wt copy of the *BP3410* gene including the native promoter and the Mn-responsive riboswitch. As controls, wt and Δ*BP3410* strains carrying an empty vector were grown together with the complemented strain in SS medium supplemented with 200 μM Mn. Again, in parallel with the optical density measurements, we determined the presence of the duplication within the *BP3410* gene by PCR. As shown in Fig. 4C, the wt strain (carrying a single copy of the gene) and the complemented strain (carrying multiple copies of the gene) resumed growth after 48 h of incubation, but the complemented strain reached stationary phase earlier than the wt strain. Both strains began to lose the duplication after 48 h, and after 72 h of incubation, they lost the duplication almost completely (Fig. 4D). We wondered whether the presence of the larger product in PCR samples of the complemented mutant indicates that some resister cells carry a mixed population of plasmid-borne copies of the *BP3410* gene that either have or have not resolved the duplication. Therefore, we plated the complemented mutant in three biological replicates on agar plates containing 1 mM Mn, and we randomly selected 30 resister colonies (10 clones per replicate) for PCR analysis. All resister clones contained only the functional copy of the *BP3410* gene (Fig. 4E). We also plated both the wt strain carrying empty plasmid and the complemented Δ*BP3410* strain in biological triplicates on agar plates containing 1 mM Mn and enumerated the colonies to determine the rate of resister formation. The complemented strain formed resister cells at more than 50-fold higher frequency (8.60 ± 1.85 × 10^−5^) than the wt strain (1.45 ± 0.58 × 10^−6^). These experiments demonstrated that ectopic expression of the *BP3410* gene complements the growth defect of the Δ*BP3410* mutant and contributes to more rapid adaptation to toxic Mn concentrations.

### Despite its homology with calcium:cation antiporters, the BP3410 protein exports manganese.

**FIG 5:**
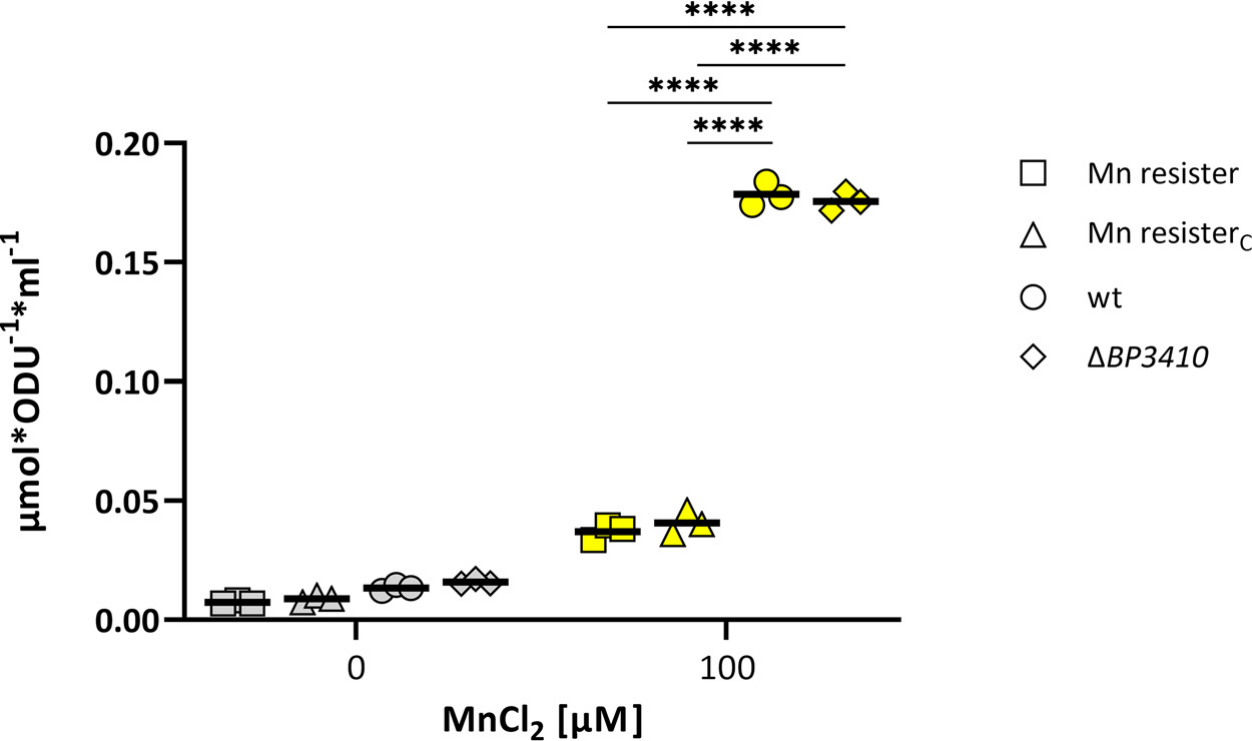
Cells producing functional BP3410 protein export manganese. Three biological replicates of wt, Δ*BP3410*, Mn resister, and Mn resister_C_ strains were cultured either in the absence (gray symbols) or in the presence (yellow symbols) of 100 μM Mn for 8 h. The amount of intracellular Mn was measured by ICP–MS/MS. The dot plot shows individual data points for all strains, and the horizontal black lines represent mean values. Statistical analysis was performed using an unpaired one-tailed *t* test; ****, *P* < 0.00005. ODU, optical density units.

Based on our results, we concluded that B. pertussis produces a nonfunctional Mn exporter, BP3410, that carries a deleterious duplication, and that this alteration prevents growth of the pathogen in the presence of toxic Mn concentrations. Consistent with the annotation of BP3410, the scan of the exporter sequence against the Pfam database identified two domains (E value of 4.4 × 10^−13^ for the N-terminal domain and E value of 2 × 10^−8^ for the C-terminal domain) characteristic of calcium:cation antiporters. In addition, searching the PDB database unveiled that the closest crystallized homolog of BP3410 (38.3% amino acid sequence similarity) is the calcium:cation antiporter of Methanocaldococcus jannaschii (44). We hypothesized that, similar to the *M. jannaschii* antiporter, the B. pertussis protein contains 10 transmembrane helices evenly distributed between two domains and that each domain contains a conservative α-repeat region, which, in complex of both domains, governs the passage of ions. When we mapped the position of the duplicated 10-amino-acid sequence in the *M. jannaschii* protein structure, we found that this duplication is located directly in the α-repeat region (data not shown) and therefore would drastically disrupt domain symmetry and most likely impair exporter function. Although these *in silico* analyses suggested that BP3410 shares significant homology with the calcium:cation antiporter superfamily, our experimental data suggested that expression and functionality of the *BP3410* gene are tied to the presence of Mn. To prove the affinity of the BP3410 exporter for Mn, we used inductively coupled plasma tandem mass spectrometry (ICP-MS/MS). We reasoned that in contrast to Mn resisters (both evolved and constructed), the strains carrying a nonfunctional copy of the *BP3410* gene (wt strain and Δ*BP3410* mutant) should accumulate Mn in the presence of this metal. Strains were grown overnight in the absence of Mn and diluted to an OD_600_ of 0.2, and then the cultures were divided and grown for 8 h in either the absence or presence of 100 μM Mn (a concentration that did not inhibit the growth of strains lacking the functional BP3410 exporter during the 8-h cultivation). Consistent with our assumption, ICP-MS/MS analysis revealed that wt and Δ*BP3410* cells contained approximately 5-fold more Mn than either type of Mn resister (Fig. 5).

### Compared with wt cells producing the nonfunctional exporter, Mn resister_C_ cells display impaired growth under oxidative stress.

**FIG 6:**
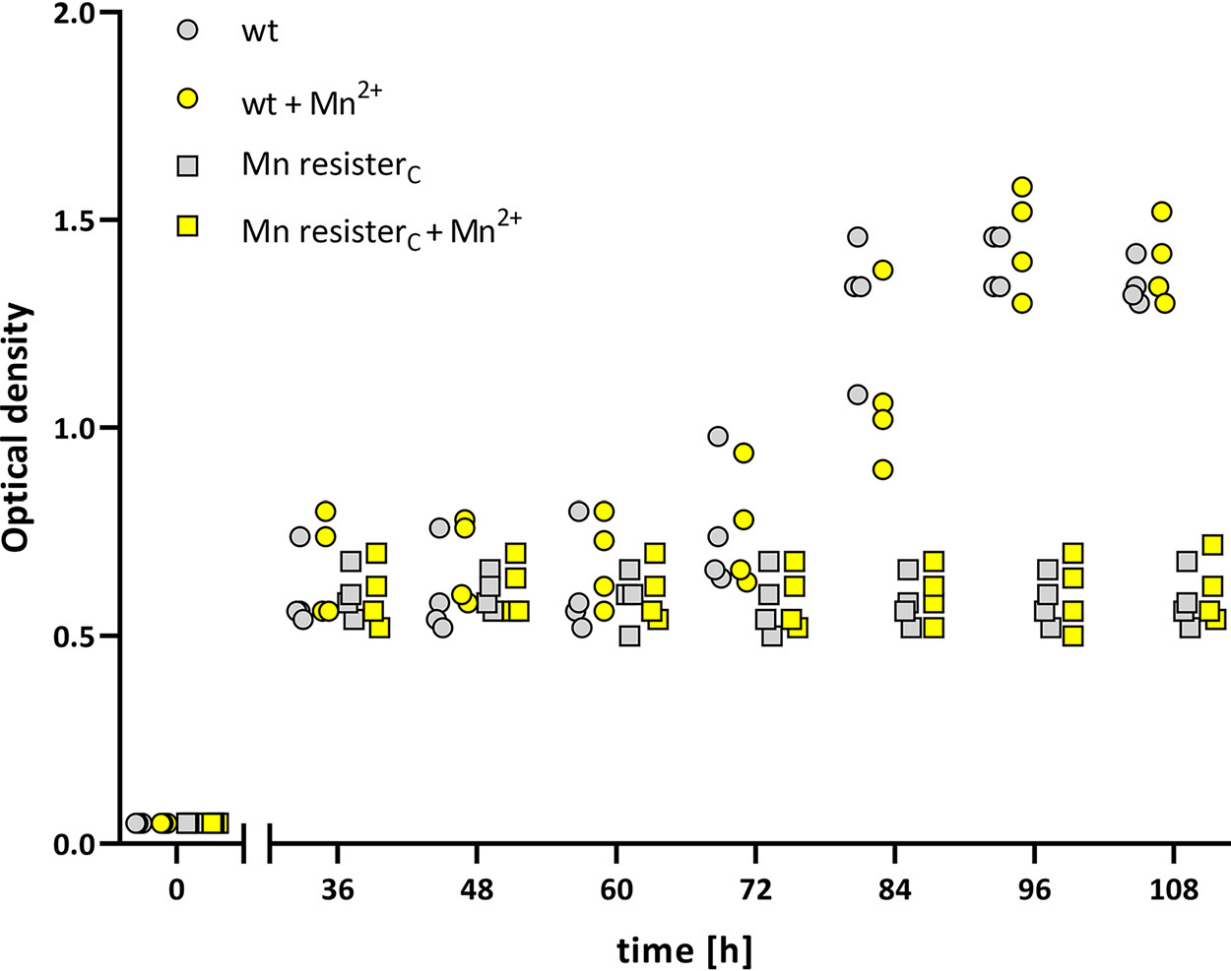
Mn resister cells do not resist oxidative stress. Four biological replicates of wt (circles) and Mn resister_C_ strains were passaged 5 times in low-iron SS medium and then pregrown for 8 h in low-iron SS medium in the absence (gray symbols) or presence (yellow symbols) of 100 nM Mn. Cultures were then treated with 70 μM dipyridyl and 0.9 mM paraquat, and after an initial period of 36 h, growth was monitored every 12 h by OD_600_ measurements. The dot plot shows the individual data points for all strains.

Given the important role of Mn in the adaptation of bacterial pathogens to the host environment, we wondered whether the deleterious duplication that renders the Mn exporter BP3410 nonfunctional confers an advantage to B. pertussis cells. We speculated that the decay of the exporter and the resulting accumulation of Mn within the cytosol favor the survival of the pathogen under oxidative stress. Therefore, to prove the concept, we tested the growth characteristics of B. pertussis strains cultured in the presence of paraquat, a known inducer of oxidative stress (45). In addition, we used iron-limited conditions because the iron-dependent repressor Fur inhibits Mn import (29), and iron deficiency mimics the conditions that prevail during infection *in vivo*. Therefore, cultures of wt and Mn resister_C_ strains were passaged several times in the iron-limited SS medium and then incubated in the presence or absence of 100 nM Mn (a concentration that is in the range of Mn concentrations in human blood [46]) for 8 h to allow Mn accumulation. Cultures were then washed with PBS containing 10 mM EDTA and diluted in SS medium supplemented with paraquat and the iron chelator dipyridyl. Growth of all cultures was monitored for 108 h, but optical density was not measured for the first 36 h, as all strains exhibited similar growth during this period (data not shown). As shown in Fig. 6, the optical density of both strains did not change substantially until 60 h of cultivation. Around this time point, however, the wt strain resumed growth, in contrast to the resister strain. Surprisingly, the resumption of growth of the wt strain was not dependent on prior exposure to Mn.

## DISCUSSION

B. pertussis represents a respiratory pathogen of humans that is continuously adapting to its exclusive host (36, 37, 47). During this process, B. pertussis underwent a global expansion of insertion elements that resulted in large genome rearrangements (change in gene order), genome reduction (loss of genes), and genome decay (loss of gene functionality) (37–39, 48). Such an evident and substantial genome reorganization process is usually associated with an evolutionarily recent change in the niche and resulting host restriction (49). Gene inactivation, primarily by insertion of massively expanded IS*481* elements, resulted in the formation of hundreds of pseudogenes within the B. pertussis genome (37). To date, the inactivation of B. pertussis genes has resulted in permanent loss of their function. Here, we report the first example of a decayed gene whose functionality can be fully restored. Our data demonstrate that the Mn exporter gene *BP3410*, which is controlled by an Mn-sensing riboswitch, is inactivated in wt cells by a 30-bp tandem duplication and thereby encodes a defective protein. However, exposure of wt cells to toxic levels of Mn resulted in formation of resister cells lacking the deleterious duplication. Loss of the duplication restored the functionality of the BP3410 exporter, as evidenced by significantly reduced intracellular Mn levels in resister cells compared with wt cells.

Our results proved that resumption of growth of the wt strain and the complemented mutant in the presence of toxic Mn concentrations correlated with the loss of duplication of the chromosome and the plasmid-borne gene, respectively. Notably, ectopic expression of the *BP3410* gene led to increased frequency of resister formation, indicating that adaptation to Mn stress is a stochastic and gene dose-dependent event. However, PCR analysis of resisters formed from complemented Δ*BP3410* strains indicated that all plasmid-borne copies of the *BP3410* gene resolved duplication, proposing that loss of duplication is not purely of a stochastic nature. The exact nature of this unique process that ensures precise excision of the duplication region is unclear, but the length, proximity, and 100% homology of the two tandem repeats imply that this process is RecA independent (50).

It remains to be determined whether the loss-of-duplication/gain-of-function process is reversible in an Mn-restrictive environment. We tested dozens of Mn resister clones isolated from human THP-1 macrophages (Mn-limited environment), but none of them carried the inhibitory duplication (data not shown). Therefore, we speculate that loss of duplication is not reversible under *in vitro* conditions and represents a dead-end state for resister cells. Interestingly, a sequence similarity search revealed that approximately 2 % of B. pertussis isolates carry a functional copy of the *BP3410* gene (data not shown) and that these strains are randomly distributed across the phylogenetic tree reconstructed by Bart et al. (51). This observation suggests that strains resistant to toxic Mn concentration have reemerged during the evolution of B. pertussis and that the gain of *BP3410* gene function, observed in our experimental setup, represents a biologically relevant backup mechanism. The genus *Bordetella* appears to be of an environmental origin (52), and species such as B. bronchiseptica and *B. petrii* can survive outside the host and, thus, may encounter environments with elevated Mn content. Moreover, most environmental isolates of *Bordetella* sp. were collected from soil (52), and some of the isolates exhibited substantial resistance to heavy metals (53, 54).

Given the uniqueness of the duplication in B. pertussis and the general importance of Mn for virulence, we hypothesized that inactivation of the Mn exporter specifically contributes to the survival of B. pertussis within the human host. Similarly, a homology search against the GenBank database showed that human *B. parapertussis* isolates carry a frameshift mutation in the *BPP3560* gene, a *BP3410* homolog. This observation supports our hypothesis that the pseudogenization of Mn exporters arose from adaptation to humans. Moreover, nearly 70 % of *Neisseria gonorrhea* strains carry a frameshift mutation within the Mn exporter MntX (17). It has been reported that this pathogen uses Mn as a quenching substance to deal with oxidative stress (55). By analogy, we reasoned that the nonfunctional Mn exporter might also help B. pertussis cells to retain the acquired Mn within the cytosol to cope with oxidative stress. Indeed, our experiments demonstrated that compared with the wt strain, the resister cells carrying a functional exporter displayed impaired growth under oxidative stress *in vitro*. Intriguingly, pretreatment with Mn had no effect on the growth of paraquat-treated wt cells. We presume that the growth medium contained trace amounts of Mn, present as an impurity in other components of the medium, which allowed B. pertussis to resist oxidative stress *in vitro* independent of additional Mn supplement. Notably, compared with the *BP3410_mutP_* strain, *BP3410* expression was significantly increased in the wt strain in the absence of Mn supplement. This finding indicated that the exporter gene is expressed to some extent even in the absence of Mn and, thus, would dictate unfavorable Mn export even under Mn-limited conditions during infection. We speculate, in the context of nutritional immunity, that “leaky” expression exposed the exporter to selection pressure that led to its decay.

Notably, we have discovered other links that document the importance of adequate Mn homeostasis for the physiological fitness of B. pertussis. One of the steps in the biosynthesis of heme, the oxidative decarboxylation of coproporphyrinogen III to protoporphyrinogen IX, is catalyzed by two isozymes, HemN and HemF (56). In contrast to HemN, HemF requires Mn as a cofactor, and *hemF* expression is induced upon oxidative stress in E. coli (57). We noticed that the *hemN* gene is nonfunctional in B. pertussis, as its coding sequence is interrupted by an IS*481* element, making heme biosynthesis in this pathogen most likely Mn dependent. Our ongoing experiments (B. Vecerek, unpublished data) also have shown that intracellular B. pertussis cells strongly upregulate the ATP-binding cassette importer (encoded by the *BP3080* to *BP3082* genes) during infection of human THP-1 macrophages. This transporter shares substantial homology with the Mn importer SitABCD of Salmonella enterica serovar *Typhimurium* (58).

Collectively, our results demonstrate that speciation of B. pertussis was accompanied by inhibitory duplication within the Mn exporter and indicate that the inactivation of the exporter represents an adaptive mechanism that maintains sufficient levels of cytosolic Mn, thereby assisting the pathogen in resistance to stress and survival within the human host.

## MATERIALS AND METHODS

### Bacterial strains and growth conditions.

Bordetella pertussis Tohama I strain (59) and its derivatives (see Table S1 in the supplemental material) were grown on Bordet-Gengou agar (BGA) plates supplemented with 15% sheep blood for 3 days at 37°C prior to inoculation into liquid medium. For liquid cultures, bacteria were grown in Stainer-Scholte (SS) medium (42) supplemented with 0.1% cyclodextrin (Sigma-Aldrich) and 0.5% Casamino Acids (Difco) in an Innova 43 orbital incubator (Eppendorf) at 37°C and 160 rpm. When required, SS medium was supplemented with various concentrations of manganese chloride (Sigma-Aldrich) as specified in figure legends. Strains bearing the pBBRMCS1 vector were grown on BGA plates supplemented with chloramphenicol (10 μg ml^−1^; Sigma-Aldrich).

10.1128/mBio.01902-21.2 TABLE S1 Strains and plasmids used in the study. Download Table S1, PDF file, 0.3 MB. Copyright © 2021 Čapek et al. 2021 Čapek et al. https://creativecommons.org/licenses/by/4.0/ This content is distributed under the terms of the Creative Commons Attribution 4.0 International license.

### Construction of the mutants.

The deletions or point mutations were introduced into B. pertussis Tohama I strain chromosome as already described (60). To construct the Δ*BP3410* deletion mutant, two DNA fragments of approximately 750 bp corresponding to the upstream region (ending with the ATG codon of the *BP3410* gene and NheI site) and the downstream regions (starting with the NheI site and TGA stop codon of the *BP3410* gene) flanking the gene. Obtained PCR products were ligated via an NheI site, and the ligation mixture was used as a template to create an approximately 1.5-kb PCR product containing the intended deletion. In the resulting product, the start and stop codons of *BP3410* separated by the NheI restriction site created a markerless in-frame deletion. A similar approach was applied to construct the *BP3410_mutP_* strain, carrying the mutated −10 region within the *BP3410* promoter. DNA fragments of approximately 750 bp adjacent to the mutated site were amplified using mutagenic primers containing the NheI site and then ligated via the NheI site. The ligation mixture was used to amplify the 1.5-kb product, in which the sequence TGCTACAAT was replaced by the NheI restriction site GCTAGC. To construct the *BP3410_mutGG_* strain, where two G residues within the riboswitch were replaced by A residues, the regions upstream and downstream of the mutation site were amplified using primers carrying the mutagenic sequence. Resulting PCR products were joined by an overlap PCR, yielding an ≈1.5-kb PCR product containing the planned mutation. To construct the Mn resister_c_ strain, the *BP3410* gene lacking the 30-nt duplication was directly amplified using the DNA of the evolved Mn resister strain as a template. In all cases, final PCR products were ligated into the allelic exchange plasmid pSS4245 and cloned in the E. coli XL1-Blue strain. Finally, the resulting recombinant plasmid was transformed into the E. coli SM10 strain (donor strain) and transferred to B. pertussis Tohama I (recipient strain) by conjugation, as described elsewhere (61). After two recombination events, the strain carrying the desired mutation was obtained. All deletions and point mutations were confirmed by DNA sequencing.

To complement the deletion of the exporter gene, the plasmid carrying the *BP3410* gene, including its promoter region, was constructed as follows. The gene was amplified using forward and reverse primers containing SacI and XhoI restriction sites, respectively. The PCR product was then cleaved with SacI and XhoI enzymes and inserted into the corresponding sites of the plasmid pBBRMCS1. Resulting plasmid was transferred to B. pertussis Δ*BP3410* recipient strain by conjugation. The plasmids used in this study and primers used for the construction of the mutants and quantitative PCR are listed in the Tables S1 and S2, respectively.

### Determination of manganese toxicity by disc diffusion assays.

Strains were inoculated from BGA plates into SS medium and grown overnight for ≈20 h, until the cells reached late exponential phase (optical density at 600 nm [OD_600_], ≈1.5 to 2). The next day, the OD_600_ value of bacterial cultures was adjusted to 2, and 100 μl of the suspension was spread on BGA plates. Next, 6-mm filter paper discs (Oxoid) were placed into the center of the dish and immediately soaked with 10 μl of 100 mM MnCl_2_. Plates were incubated for 4 to 5 days until the clear inhibition zones were formed.

### Determination of frequency of resister formation.

Strains were inoculated from BGA plates into SS medium and grown overnight for ≈20 h until the cells reached late exponential phase (OD_600_, ≈1.5 to 2). Strains bearing pBBRMCS1 derivatives were inoculated from BGA plates supplemented with chloramphenicol (10 μg ml^−1^). The next day, the bacterial cultures were either appropriately diluted and spread on BGA plates without Mn or spread undiluted on BGA plates supplemented with 1 mM Mn. CFU were enumerated from both types of plates, and frequency of resister formations for each strain was calculated as a ratio between CFU of formed resisters recovered from plates with Mn and the total number of CFU counted on plates without Mn. Frequencies were calculated as means and standard deviations from at least four independent experiments.

### Monitoring of Mn resister formation in liquid cultures.

Strains were grown in SS medium supplemented with 200 μM MnCl_2_. In parallel to optical density measurements, samples for isolation of the genomic DNA were taken every 24 h. Isolated genomic DNA, serving as a template, and primer pair flanking the 30-nt duplication within the *BP3410* gene (Table S2) were used to monitor the loss of the duplication. PCR was performed with Herculase II fusion DNA polymerase (Agilent Technologies) in 20-μl reaction mixtures containing 0.1 μl of the polymerase, 5 nmol each deoxynucleoside triphosphate, 4% dimethyl sulfoxide (Sigma-Aldrich), and 5 pmol each forward and reverse primer. Each reaction consisted of an initial step at 98°C for 2 min, followed by 35 cycles of 98°C for 20 s, 62°C for 30 s, and 72°C for 30 s and finishing with an amplification step at 72°C for 5 min. The amplicons were visualized using Midori Green stain (Nippon Genetics) on 3% agarose gel. The PCR products of 329 bp and 299 bp indicated the presence and absence of the duplication, respectively.

10.1128/mBio.01902-21.3 TABLE S2 Primers used in this study. Download Table S2, PDF file, 0.3 MB. Copyright © 2021 Čapek et al. 2021 Čapek et al. https://creativecommons.org/licenses/by/4.0/ This content is distributed under the terms of the Creative Commons Attribution 4.0 International license.

### RT-qPCR analysis.

Cells were grown in biological triplicates to mid-exponential phase and then incubated for 60 min in the presence or absence of 400 μM MnCl_2_. Next, cells were mixed with stop solution (95% ethanol, 5% phenol at 4:1 ratio), pelleted, and stored at −80°C. RT-qPCR analysis of *BP3410* transcript levels was performed as described earlier (60). Briefly, bacterial pellets were suspended in TE buffer (10 mM Tris, 1 mM EDTA, pH 8.0) containing 1 mg ml^−1^ lysozyme (Sigma-Aldrich), and total RNA was isolated from lysed cells using TRI reagent (Sigma-Aldrich) according to the manufacturer’s protocol. Removal of DNA was achieved by treatment of samples with DNase I (Sigma-Aldrich) for 15 min at room temperature. After organic extraction, the RNA was precipitated with 75% ethanol and dissolved in RNase-free water. RNA concentration was measured for each sample in technical triplicates using spectrophotometer DS-11 (DeNovix). Isolated RNA (600 ng) was reverse transcribed into cDNA using random hexamers and M-MLV reverse transcriptase (Promega) at 37°C in a 25-μl reaction mix according to the manufacturer’s instructions. RT-qPCRs were performed in at least three technical replicates per sample on a Bio-Rad CFX96 instrument using SYBR green JumpStart *Taq* ReadyMix (Sigma-Aldrich), 4 pmol of each primer, and 2 μl of 10× diluted cDNA in a 20-μl reaction. Each reaction consisted of an initial step at 95°C for 2 min, 40 cycles of 95°C for 15 s, 65°C for 30 s, and 72°C for 30 s, followed by melting curve recording. The *rpoB* gene was used as the reference gene and relative gene expression was determined using delta-delta threshold cycle method (62).

### ICP-MS/MS analysis.

Throughout this experiment, B. pertussis strains were cultured in SS medium that was not supplemented with iron. Biological triplicates of overnight (≈18 h) cultures of each strain were diluted the next day to an OD_600_ of 0.2 in 20 ml of the fresh medium and split into two flasks, each containing 10 ml of culture. To the first culture, MnCl_2_ was added to a final concentration of 100 μM, while the second culture, lacking Mn, served as a control. All cultures were then grown in an orbital incubator for 8 h to allow for Mn accumulation. The OD_600_ was measured in all cultures to verify that manganese did not impose toxic effect on bacterial growth (i.e., OD_600_ of Mn-treated cultures was similar to that of control cultures). Next, cells were washed twice in phosphate-buffered saline (PBS; pH 7.4) containing 10 mM EDTA and once with PBS only. Finally, cells were suspended in PBS and normalized to the OD_600_ so that after centrifugation, each pellet contained cells corresponding to 2 OD_600_ units. Bacterial pellets were decomposed in 1 ml of 65% HNO_3_ (Honeywell) for 15 min at 80°C. An aliquot of 0.9 ml was then 10× diluted using 8.1 ml of deionized water. Determination of the intracellular Mn levels by ICP-MS/MS with triple quadrupole was carried out using an Agilent 8900 spectrometer equipped with an SPS 4 autosampler and ISIS3 sample introduction system. Mn was detected at *m/z* 55 in single-quad He mode (He, 4.5 ml min^−1^) and at *m/z* 55→55 and 55→71 in MS/MS O_2_ reaction mode (O_2_ 40% gas flow rate) to ensure interference-free analysis. Yttrium (*m/z* 89 and 89→103) was used as an internal standard to correct for sensitivity drifts. Quantification was performed using six-point external calibration (0.125 to 4.0 μg liter^−1^ Mn). Calibration standards were prepared from Mn standard solution (1 g liter^−1^ Mn; Sigma-Aldrich) in 10× diluted HNO_3_. Results were processed using Agilent MassHunter 4.5 and Microsoft Excel software, and the result for each biological replicate is an average from two parallel ICP-MS/MS measurements at three tune modes. Three blank samples without bacteria were processed simultaneously with the experimental samples to determine background concentrations of Mn, which then were subtracted from values obtained with experimental samples.

### Multiple-sequence alignment of calcium:cation antiporters.

The alignment of BP3410 exporter with homologous proteins was performed using complete BP3410 sequence as a query in a gapped BLAST search, which is integrated as a first step of PSI-BLAST (63); no further iterations were necessary. When needed, BLOSUM matrix was reduced to 45 in order to detect distantly related proteins. Representative sequence of each species with the highest score was included in the alignment. Additionally, the presence of two calcium:cation antiporter domains was verified by Pfam database search. NCBI accession numbers for each sequence were WP_010931461.1 (B. pertussis), WP_003820973.1 (B. bronchiseptica), WP_115522187.1 (*A. veronii* AG_5.28.6), WP_115522187.1 (*A. veronii* VCK_1), WP_115522187.1 (*A. veronii* BIOO50A), NEY27906.1 (E. coli), WP_164818558.1 (C. perfringens), WP_010869583.1 (*M. jannaschii*), PNW75261.1 (C. reinhardtii), NP_010155.1 (S. cerevisiae), OAO96401.1 (*A. thaliana*), NP_001104467.2 (D. melanogaster), XP_637590.1 (D. discoideum), EDL28124.1 (*M. musculus*), XP_018108579.1 (*X. laevis*), NP_499146.2 (C. elegans), AAS76476.1 (*D. rerio*), XP_025006903.1 (*G. gallus*), and AAM76071.1 (H. sapiens). Sequences were aligned by MUSCLE algorithm (64) built in MEGA 7.0.21 software (65) using the neighbor-joining clustering method, and the resulting alignment was refined by ESPript 3.0 (66). An exception to this procedure was the transporter Vcx1 from Saccharomyces cerevisiae, which has been included based on its known tertiary structure, as a gapped BLAST search did not return a positive result (67). The similarity between BP3410 and *M. jannaschii* exporter was calculated using the Needleman–Wunsch algorithm (68) using the BLOSUM62 scoring matrix.

### Sensitivity to oxidative stress.

In the sensitivity to oxidative stress experiment, B. pertussis cells were cultivated in low-iron SS medium (i.e., medium was not supplemented with 36 μM FeSO_4_). To reduce the cytosolic content of manganese and iron in cultivated cells, cultures were passaged every 12 h (five passages in total). For the last passage, each culture was diluted to an OD_600_ of 0.2 into 20 ml of the fresh medium and split into two flasks, each containing 10 ml of culture. To the first culture, MnCl_2_ was added to a final concentration of 100 nM, while the second culture, lacking Mn, served as a control. All cultures were then grown for an additional 8 h to allow the cells to accumulate manganese. The cells were washed twice with PBS (pH 7.4) containing 10 mM EDTA and once with fresh low-iron SS medium. Finally, cells were resuspended in 1 ml of fresh SS medium and diluted to an OD_600_ of 0.05 in 8 ml of fresh low-iron SS medium containing 70 μM dipyridyl (Sigma-Aldrich) to chelate the remaining iron and 0.9 mM paraquat (Sigma-Aldrich) to induce the oxidative stress. Cultures were grown for 36 h, and then the optical density of cultures was monitored every 12 h.

### Data availability.

All study data are included in the article and/or supporting information.
