# Supplementary material for: A Unique Reverse Adaptation Mechanism Assists Bordetella pertussis in Resistance to Both Scarcity and Toxicity of Manganese
Source: mBio. 2021 Oct 26;12(5):e01902-21. doi: 10.1128/mBio.01902-21 (PMC8546581; doi:10.1128/mBio.01902-21)
Supplement: FIG S1 [file mbio.01902-21-sf001.pdf]

```
                                                                          1
Bordetella_pertussis_Tohama_I     .......................................MFIT.................
Aeromonas_veronii                 .................MHASRASHVGSIGYSARLAVSIMLIT.................
Bordetella_pertussis_Mn_resister  .......................................MFIT.................
Bordetella_bronchiseptica         .......................................MFIT.................
Escherichia_coli                  .........................................MY.................
Clostridium_perfringens           ............................................................
Methanocaldococcus_jannaschii     .......................................MLIL.................
Chlamydomonas_reinhardtii         .....MSAQRKNAISVVKADARSRLEAAIDENDEQAYDEALLGAANGGVEGNGFARQSAG
Dictyostelium_discoideum          .....................................MTPVIT.................
Saccharomyces_cerevisiae          ..........MDATTPLLTVANSHPARNPKHTAWRAAVYDLQY.................
Caenorhabditis_elegans            .............MRIHFFAFLIIILSLVGCDGWQADVGKLVRKWNNQRTIDGNDIEKCI
Arabidopsis_thaliana              ...........MASLFSSRLSSQSLSLLINIFFIFLIFLHFASQNPPPSGSIQTLNSFAG
Drosophila_melanogaster           .............MFFDWSSTDFKPNGNEWEYYPFAYRHDMRY.................
Danio_rerio                       .........MRTDVFLQRRKRRDVLLSIIALLLLIFAIVHLVFCAGLSFQGSSSA.....
Xenopus_laevis                    MEKGSVALNVGRRPFGKKRLPCRAPLGSFALFLIVCGTVYLVNQVATTLVVRGTQ.....
Gallus_gallus                     MELDGAPLARPKTMLLLLRKVRKRREMLLQQLGFICAILFFVWCMSSLLSRRGQESSIAD
Mus_musculus                      ............................................................
Homo_sapiens                      ..........................MLPQQVGFVCAVLALVCCASGLFGSLGHKTASAS
```

```
                                                                                           10
Bordetella_pertussis_Tohama_I     .....................................................LLLFFLS
Aeromonas_veronii                 .....................................................LLLFLVS
Bordetella_pertussis_Mn_resister  .....................................................LLLFFLS
Bordetella_bronchiseptica         .....................................................LLLFFLS
Escherichia_coli                  ...................................................QTITFFFLS
Clostridium_perfringens           .....................................................MNYIFLI
Methanocaldococcus_jannaschii     ....................................................GVGYFLLG
Chlamydomonas_reinhardtii         AGA..........RTTSGRNSITGDRTLSRGSILSVAPKWFV..RDYMALKEIVFSSYIN
Dictyostelium_discoideum          ........................................................AFLS
Saccharomyces_cerevisiae          ....................................................ILKASPLN
Caenorhabditis_elegans            PDDGEQCTIQAGWNSTQVCEYVKCNDDNCEGGGYVLWTRYVECASSPAVRIILIITGVIY
Arabidopsis_thaliana              GDSDSCSGGLASLDDHRSKCSYIRSQSKCGPQGYIDYLKIFFCIFGQSPVLGHLVLSAWL
Drosophila_melanogaster           ......................DIDARNCTLPAIVEFPNLM.....RQKSWIAIVVSTLV
Danio_rerio                       .............RVRRDL....ENASECVQPQSSEFPEGFF..TVQERKDGGILIYFMI
Xenopus_laevis                    .............RIRRDI....ENETLCIASPSSEFPEDFF..TEQERKQGGLIIHFLV
Gallus_gallus                     TRVPSETWRN...RILMALANGTHEGKNCTEPAIHEFPEDIF..TNKERQQGGVLLHIIA
Mus_musculus                      ............................................................
Homo_sapiens                      KRVLPDTWRN...RKLMAPVNGTQTAKNCTDPAIHEFPTDLF..SNKERQHGAVLLHILG
```

```
                                             20                           30         40
Bordetella_pertussis_Tohama_I     AAIIYF....ACEF...............FVN...GVEWVGHRFKLGA.TATGTVLAAFG
Aeromonas_veronii                 AVTIYL....ACEY...............FVN...GIEWTGHHFKLSQ.SATGSLLAALG
Bordetella_pertussis_Mn_resister  AAIIYF....ACEF...............FVN...GVEWVGHRFKLGA.TATGTVLAAFG
Bordetella_bronchiseptica         AAIIYF....ACEF...............FVN...GVEWVGHRFKLGA.TATGTVLAAFG
Escherichia_coli                  AIAIYL....ACEC...............FVN...GIEWFGRRLNIGA.TAVGSVLAAFG
Clostridium_perfringens           IGFLLL...IKGADL..............FVD...GASGIAKKLGVPA.VIIGLTIVSLG
Methanocaldococcus_jannaschii     LILLYYGSDW...................FVL...GSERIARHFNVSN.FVIGATVMAIG
Chlamydomonas_reinhardtii         LMLLCAPLGWASAYFKWGAVATFSLNFLALIPLALILGDITEDLAVRFGDVVGGLINATF
Dictyostelium_discoideum          FAFLCF.ASWCVGE.....................GGEILGKKYDA...SIIGGLIIAWL
Saccharomyces_cerevisiae          FLLVFVPLGLIWGHFQLSHTLTFLFNFLAIIPLAAILANATEELADKAGNTIGGLLNATF
Caenorhabditis_elegans            MLVLFIMVSSAADDF..............FSP...SISSIVAHLRISE.SVAGVTFMAFG
Arabidopsis_thaliana              FVLFYL.LGDTAASY..............FCP...SLDSLSKVLKLSP.TMAGVTLLSLG
Drosophila_melanogaster           SMYLFVILAIVCDDY..............LVP...AMERLCYTLRMTY.DVAGATFLAAS
Danio_rerio                       IFYMLLSVSIVCDEY..............FLP...SLEVISERLGLSQ.DVAGATFMAAG
Xenopus_laevis                    ILYMFLAVSVVCEYY..............FIP...SLEVISERLGLSQ.DVAGATFMAIG
Gallus_gallus                     ALYMFYALAIVCDDF..............FVP...SLEKICERLHLSE.DVAGATFMAAG
Mus_musculus                      ...MCMAISIVCDKY..............FLP...SLEIISDSLGLSQ.DVAGATFMAAG
Homo_sapiens                      ALYMFYALAIVCDDF..............FVP...SLEKICERLHLSE.DVAGATFMAAG
```

```
                                  50        60        70        80        90      100
Bordetella_pertussis_Tohama_I     TALPESAVTFMAVVFGTTPEQKDIGVGAAMGGPLVLATLAYAVVGLTLARTRRAGSSQVE
Aeromonas_veronii                 TALPECIVTLTALLMGNTTAQQEIGIGAALGGPLVLATIGYGAVGLTLLLCMGRTE....
Bordetella_pertussis_Mn_resister  TALPESAVTFMAVVFGTTPEQKDIGVGAAMGGPLVLATLAYAVVGLTLARTRRAGSSQVE
Bordetella_bronchiseptica         TALPESAVTFMAVVFGTTPEQKDIGVGAAMGGPLVLATLAYAVVGLTLARTRRAGSSQVE
Escherichia_coli                  TALPESTVTFMAVVFGSTPEQKNIGVGAAMGGPLVLATLAYAVVGLALICHHRRSNRKS.
Clostridium_perfringens           TSAPELAVSISASLKG....SNDITMGNVLGSNLFNLLAALGCTAIV.......AP....
Methanocaldococcus_jannaschii     TSLPEILTSAYASYMH....APGISIGNAIGSCICNIGLVLGLSAII.......SP....
Chlamydomonas_reinhardtii         GNVVEVMVSLFALFKN....LFTVVAASLLGSILSNLLLVMGCSFLL.......GG....
Dictyostelium_discoideum          NTAPEAIFFITALTSD....NTRFAVGAVSGSSIVVCTVALGCCIVL.......GT....
Saccharomyces_cerevisiae          GNAVELIVSIIALKKG....QVRIVQASMLGSLLSNLLLVLGLCFIF.......GG....
Caenorhabditis_elegans            NGAPDVFGAIASVLSSPTP.KADLALGELFGAGLFVTTMV...LAVTI......FT....
Arabidopsis_thaliana              NGAPDLFSSVVSFTRSN...NGDFGLNSILGGAFFVSSFVVGTICVLI......GS....
Drosophila_melanogaster           TSAPELFVNFVGTFVT....NGDIGLGTIVGSSVFNILVIAGVCGIF.......T.....
Danio_rerio                       SSAPELVTAFLGVFVT....KGDIGVSTIMGSAVYNLLCICAACGLL.......SS....
Xenopus_laevis                    SSAPEFVTVFLGVFVT....KGDIGVSTIVGSAVYNLLGICAACCLL.......SS....
Gallus_gallus                     SSTPELFASVIGVFIT....HGDVGVGTIVGSAVFNILCIVGVCGLF.......AG....
Mus_musculus                      SSAPELVTAFLGVFIT....KGDIGISTILGSAIYNLLGICAACGLL.......SN....
Homo_sapiens                      SSTPELFASVIGVFIT....HGDVGVGTIVGSAVFNILCIIGVCGLF.......AG....
                                      ★
```

```
                                  110          120       130       140         150
                                   .            .         .         .           .
Bordetella_pertussis_Tohama_I     AAINADQRR...LARDQAWFMGVFVFKVALGLLAFAWKP..WLGLLFLAVYGLYV.....
Aeromonas_veronii                 RAIDGEVQRE..IGRDQRWFMGIFLVATALGLVAFTGKS..WLAPLFLLVYLVYV.....
Bordetella_pertussis_Mn_resister  AAINADQRR...LARDQAWFMGVFVFKVALGLLAFAWKP..WLGLLFLAVYGLYV.....
Bordetella_bronchiseptica         AAINADQRR...LARDQAWFMGVFVFKVALGLLAFAWKP..WLGLLFLAVYGLYV.....
Escherichia_coli                  AKIRVDYAR...MSHDQAAFLAVFFVKVALGLIVFTLKP..WLGVLFLVAYALYV.....
Clostridium_perfringens           LVIKKHIIKNDFIVNLIATFILYVFT..FTGLIGSKNSLSRIDGGILVLMCITYII....
Methanocaldococcus_jannaschii     IIVDKN......LQKNILVYLLFVIFAAVIGIDGFS....WIDGVVLLIL...FII....
Chlamydomonas_reinhardtii         MYHSVQTFN...AMSNRACSSLLMLA..CIGIS.IPSA.....ASMLLVTDPALRA....
Dictyostelium_discoideum          RNRKGGSFQ...LQPPVKKQCLILL.....LSLL.IP......LFLSLVGYNVFFG....
Saccharomyces_cerevisiae          YNRVQQTFN...QTAAQTMSSLLAIA..CASLL.IPAA...FRATLPHGKEDHFIDGK..
Caenorhabditis_elegans            RPFKAEVFS...SIRDIAFYLVALAF.LAFCFV.YYDHVEIWMPITFLGVYLIYVCTVIL
Arabidopsis_thaliana              RDVSIDRNS...FIRD.VVFLLVALC..CLGLIIFIGKVTIWVALCYLSIYLLYVG....
Drosophila_melanogaster           QPTKLDWWP...VTRDTAWYLIAIAS..LTYVL.WDSLVMWYEAFALLLLYISYVI....
Danio_rerio                       AVGRLSCWP...LFRDCVAYSISVAA..VIAII.SDNRVYWYDGACLLLVYGVYVA....
Xenopus_laevis                    SISRLTCWP...LFRDCVAYAISVAA..VIAIT.FDNRIYWYESASLLLIYGIYIV....
Gallus_gallus                     QVVCLTQWA...VFRDSVYYTLSVII..LIVFI.HDEKIEWWESLVLIIMYSFYIL....
Mus_musculus                      MVSTLSCWP...LFRDCAVYAVSVGA..VFGII.FDNRIYWYEGAGLLLIYGLYVL....
Homo_sapiens                      QVVRLTWWA...VCRDSVYYTISVIV..LIVFI.YDEQIVWWEGLVLIILYVFYIL....

                                          160                                       170
                                           .                                         .
Bordetella_pertussis_Tohama_I     ........KRELTREEE................................CLDCEDLE...
Aeromonas_veronii                 ........RRELKQSDS.................................HEVELPE...
Bordetella_pertussis_Mn_resister  ........KRELTREEE................................CLDCEDLE...
Bordetella_bronchiseptica         ........KRELTREEE................................CLDCEDLE...
Escherichia_coli                  ........WREIRNSDT................................APEEEMIE...
Clostridium_perfringens           ..YL....IYTVKRSNK...........................KEDEVALTAEIED...
Methanocaldococcus_jannaschii     ..YL....RWTVKNGSA....................................EIEE...
Chlamydomonas_reinhardtii         ..DW....ILDVSRGTA.........................VIMLICYACYLTF.....
Dictyostelium_discoideum          ........IFG.............................IIYYLIFIGYSLFHKLPE...
Saccharomyces_cerevisiae          ........ILELSRGTS.........................IVILIVYVLFLYFQ....
Caenorhabditis_elegans            SQILHNRHKKDEEKVDEVKTVDTDSLEDDDDIYVSHGHHVLHAHEVMKMEAEIEAVE...
Arabidopsis_thaliana              ..FLSVSHFFDRKK............................RMSDQILRSREDLAE...
Drosophila_melanogaster           ..QL....SFDRRIQNLV........................RHEHAESELLDEDPM...
Danio_rerio                       ..VL....CFDLKISEY......................VMQRFSPCCWCLKPRDRD...
Xenopus_laevis                    ..IM....CFDIKISKY......................VVRRFSPCCACCAEAMVE...
Gallus_gallus                     ..IM....KYNVRMQNF.FTLKSKNVGNGDTVDNEMEDGNKCYDSSCDDPSIPLLWKVKG
Mus_musculus                      ..LL....CFDTTISRH......................VMKTCSPCCPCLARAMEE...
Homo_sapiens                      ..IM....KYNVKMQAFF....................TVKQKSIANGNPVNSELEAVKE

Bordetella_pertussis_Tohama_I     .........................PLK................................
Aeromonas_veronii                 .........................PLKL...............................
Bordetella_pertussis_Mn_resister  .........................PLK................................
Bordetella_bronchiseptica         .........................PLK................................
Escherichia_coli                  .........................PLK................................
Clostridium_perfringens           ..........................................................FE
Methanocaldococcus_jannaschii     ............................................................
Chlamydomonas_reinhardtii         .........................QLK.........................THSELFK
Dictyostelium_discoideum          ......DEKKEDRD...........IEMGERKNGANEQTTAST..........TASSTES
Saccharomyces_cerevisiae          ...........................LG........................SHHALFE
Caenorhabditis_elegans            .........................PLKTWSFRGVVHDFKEHLKPW.......PSMDEWD
Arabidopsis_thaliana              ......................MGVSLLGYIAEEKLALPEKTT..........QEFKIVF
Drosophila_melanogaster           .........TREEE...........PLKGFRDHVCAKPK..............TEYNFYQ
Danio_rerio                       .........SGEQQ...........PLVGWSDDSSLRVQRRSR..........NDSGIFQ
Xenopus_laevis                    .........NTEHA...........PLLGWKEESLPVIRRHSR..........SDSGIFQ
Gallus_gallus                     VQQYGKNSVVMVDEIICSSPPKYRFPEAGLRIMITNKFGPRTRMRMASRIIINERQRLIQ
Mus_musculus                      .........RIEQQ...........TLLGWEDESQLFIRRQSR..........TDSGIFQ
Homo_sapiens                      KPQYGKNPVVMVDEIMSSSPPKFTFPEAGLRIMITNKFGPRTRLRMASRIIINERQRLIN

Bordetella_pertussis_Tohama_I     ............................................................
Aeromonas_veronii                 ............................................................
Bordetella_pertussis_Mn_resister  ............................................................
Bordetella_bronchiseptica         ............................................................
Escherichia_coli                  ............................................................
Clostridium_perfringens           EQ..........................................................
Methanocaldococcus_jannaschii     ............................................................
Chlamydomonas_reinhardtii         GDD.........................................................
Dictyostelium_discoideum          DDEDTDE.....................................................
Saccharomyces_cerevisiae          QQEEETDEV...................................................
Caenorhabditis_elegans            EMNIFQKVI.....AVLNTIPNFLFKLTIPHSEQPWSKPITLLQCLICPVFLLFCIQVCS
Arabidopsis_thaliana              EDSPKRHRSCFSVLVSIIGLPLYLPRRLTIPVVCEEKWSKPCAVVSTAIAPVLLTELYCS
Drosophila_melanogaster           ............................................................
Danio_rerio                       DDSGYSHLS.....LSLHGLNEI.................................SDEH
Xenopus_laevis                    EDSDYSQLS.....ISLSGLKEP.................................SNNP
Gallus_gallus                     SANGSSK.P.....LQNGRHQNIENGNIPVVNQEEEN...................EQDN
Mus_musculus                      EDSGYS......................................................
Homo_sapiens                      SANGVSSKP.....LQNGRHENIENGNVPVENPEDPQQNQEQQPPPQPPPPEPEPVEADF
```

```
                                                        180       190         200
Bordetella_pertussis_Tohama_I       ............................LRPRDPSPSLFWA....CAQTVLALAVIALAS
Aeromonas_veronii                   .............................APDAVIP..P.VGKV.LLQTLAALVVVALAA
Bordetella_pertussis_Mn_resister    ............................LRPRDPSPSLFWA....CAQTVLALAVIALAS
Bordetella_bronchiseptica           ............................LRPRDPSPSLFWA....CTQTVLALAVIALAS
Escherichia_coli                    ............................IRPSAIEPPMGWI....VLQIVLSLIVIAAAS
Clostridium_perfringens             ...............................NIKSP..NSTVKN.IILSIVGVIGIILGG
Methanocaldococcus_jannaschii       .............................NNDKNNP....SVVFSLVLLIIGLIGVLVGA
Chlamydomonas_reinhardtii           ..............................DDAVPM..MTVGTS.IGALTVITAIVAVCS
Dictyostelium_discoideum            .............................EDEHDEP..LYKGVF...YLFLGGLLICYFS
Saccharomyces_cerevisiae            ............................MSTISRNPHHSLSVKSSLVILLGTTVIISFCA
Caenorhabditis_elegans              ISPFPNSPGLWMYGLILSVLLTAAVLFFTELHKEPP..FYKSLISYAGFLMSIAWIYLIS
Arabidopsis_thaliana                HYSGSQRNLILYIISGSIGLIVGILAYLTTEKSHPPKKFSLVWL.LGGFTMSVTWTYMIA
Drosophila_melanogaster             ............WTWWAIKYPAELML.....ACTVP..SARSIF.FLSMISAILWISLIS
Danio_rerio                         KSVFSMPDHDLKRILWVLSLPVSTLLFVSVPDCRRP..FWKNFY.MLTFLMSAVWISAFT
Xenopus_laevis                      PSVFKMPENDLRRIIWVLSLPIITLFYLTVPDCRRK..TWKKWF.ILTFVMSAVWISAVT
Gallus_gallus                       LSPFSLPEGKMNKIKWILTWPLIFVLFCTIPNCSKP..RWESWF.MFTFILSTLWIALFS
Mus_musculus                        ...............................................QLSLSLHGLSQVS
Homo_sapiens                        LSPFSVPEARGDKVKWVFTWPLIFLLCVTIPNCSKP..RWEKFF.MVTFITATLWIAVFS
```

```
                                      210       220       230       240       250        260
Bordetella_pertussis_Tohama_I       HVFVRQIELLGLAMGASPHVAALLLAPVATELPEIMNALIWVRQGKER...LALANISGA
Aeromonas_veronii                   QLFVHQLESMGELFGLSPVLAALLLSPVATETPELMNVLIWVRQGKNR...LALANISGA
Bordetella_pertussis_Mn_resister    HVFVRQIELLGLAMGASPHVAALLLAPVATELPEIMNALIWVRQGKER...LALANISG.
Bordetella_bronchiseptica           HVFVRQIELLGLAMGASPHVAALLLAPVATELPEIMNALIWVRQGKER...LALANISG.
Escherichia_coli                    HIFISQLENIGTIMHWPPHLVALFLSPVATELPEIMNALIWVRQGKER...LALANISG.
Clostridium_perfringens             KIVVDSASAIALGLGLSEKLVGLTIVAIGTSLPELVTSLVAAKKGEND...IALGNILG.
Methanocaldococcus_jannaschii       ELFVDGAKKIALALDISDKVIGFTLVAFGTSLPELMVSLAAAKRNLGG...MVLGNVIG.
Chlamydomonas_reinhardtii           EFLTDSIEEVSAHTGISETFIGLILLPIAGNACEHLTACIVAMRNKMD...LSMGVAVG.
Dictyostelium_discoideum            KPFIDSIVSLASSWEINPILLAFFLAPIASEMPEILESISLSRKGHSQSINIAFSNLIG.
Saccharomyces_cerevisiae            DFLVGTIDNVVESTGLSKTFIGLIVIPIVGNAAEHVTSVLVAMKDKMD...LALGVAIG.
Caenorhabditis_elegans              SEVVNVVTMLGVVSRVSHEVLGLTILAWSNSIGDLIADVSVAKQG...YPRMAMAAAIG.
Arabidopsis_thaliana                QELVSLLISLGNIFGISPSVLGLTVLAWGNSLGDLIANVTVAFHGGNDGAQIALSGCYA.
Drosophila_melanogaster             YLLTWFLTILGYNLNIPDAIMGLTVLAAGTSVPEVASSYIVSKKGYGS...MAICNAIG.
Danio_rerio                         YVLVWMVTIVGETLGIPDTVMGMTLLAAGTSIPDTVASVMVAREGKSD...MAMSNIVG.
Xenopus_laevis                      YILVWMVTIVGETLNIPDTVMGLTLLAAGTSIPDTVASVIVAREGKGD...MAMSNIVG.
Gallus_gallus                       YFMVWMVTVIGYTLGIPDVIMGITFLAAGTSVPDCMASLIVARQGLGD...MAVSNTIG.
Mus_musculus                        E.........GETLGIPDTVMGLTLLAAGTSIPDTVTSVLVARKGKGD...MAISNIVG.
Homo_sapiens                        YIMVWLVTIIGYTLGIPDVIMGITFLAAGTSVPDCMASLIVARQGLGD...MAVSNTIG.
                                                                       ★                  ▲
```

```
                                         270       280       290          300         310
Bordetella_pertussis_Tohama_I       MMIQATIPSAMMIQATIPSALGIFLTPWLL.......DAPL.LAAG...GFTLAAI..LL
Aeromonas_veronii                   MMIQGTIPKAMMIQGTIPKAFCLLFTPWLL.......DGTM.MASS...LVTLLAV..GL
Bordetella_pertussis_Mn_resister    .........AMMIQATIPSALGIFLTPWLL.......DAPL.LAAG...GFTLAAI..LL
Bordetella_bronchiseptica           .........AMMIQATIPSALGIFLTPWLL.......DAPL.LAAG...GFTLAAI..LL
Escherichia_coli                    .........AMMIQATIPSALGLFFTPWLF.......DGPL.LVAG...IVTALAI..VY
Clostridium_perfringens             .........SNTFNILLI..LGLS...SVI........SPITIAASLSIDLIFLIIVTLI
Methanocaldococcus_jannaschii       .........SNIAD..IGGALAVG...SLF..........MHLPAE...NVQMAVL..VI
Chlamydomonas_reinhardtii           .........SSIQIALFAIPFAVV.VGWAT.......GHDFSLDFS.....PFSAM..VL
Dictyostelium_discoideum            .........GTITKTTLL..MGIF...CFF.......GVVKGFEWE...SPTYSLC..LI
Saccharomyces_cerevisiae            .........SSLQVALFVTPFMVL.VGWMI.......DVPMTLNFS.....TFETA..TL
Caenorhabditis_elegans              .........GQLFNLLIG..FGLP....FTIAKIQGKSISMIIN......PTYRLL..ML
Arabidopsis_thaliana                .........GPLFNTVIG..LGVP....LVISSLAEYPGVYIIPSD...NSLLETLGFLM
Drosophila_melanogaster             .........SNTFDILVC..LGLP...WLLKILI..YQQKIDIDST...ALTITTA..ML
Danio_rerio                         .........SNVFDMLC...LGLP...WFIQTVFVDVGSPVDVNSS...GLVFMSC..TL
Xenopus_laevis                      .........SNVFDMLC...LGVP...WFIKTVFVDRSSPVEVNSS...GITYTTI..SL
Gallus_gallus                       .........SNVFDILVG..LGVP...WGLQTMAIDYGSTVKINSK...GLVYSVA..LL
Mus_musculus                        .........SNVFDMLC...LGLP...WFIKTAFTNASAPIEVNSK...GLTYITI..SL
Homo_sapiens                        .........SNVFDILVG..LGVP...WGLQTMVVNYGSTVKINSR...GLVYSVV..LL
                                    ▲▲▲▲▲▲▲▲▲▲▲▲▲▲▲▲▲▲▲
```

```
                                                320              330       340
Bordetella_pertussis_Tohama_I       L...WLRFRRAAMSVPALSA......VG..GLYALFAGYLGWHFYA..............
Aeromonas_veronii                   L...WWGFRKGTMAAGRLAL......VG.....LLYLGFCGWLLVS..............
Bordetella_pertussis_Mn_resister    L...WLRFRRAAMSVPALSA......VG..GLYALFAGYLGWHFYA..............
Bordetella_bronchiseptica           L...WLRFRRAAMSVPALSA......VG..GLYALFAGYLGWHFYA..............
Escherichia_coli                    L...WQVFRRGIADGRALII......VS..GGYGLFVLLVLGYVA...............
Clostridium_perfringens             IGALIFLNKKKEKVLTKY........EG.VFLLALYIGYTVYIIMRN.............
Methanocaldococcus_jannaschii       MSLLLYLFAKYS.KIGRW........QG.ILFLALYIIAIASLRMG..............
Chlamydomonas_reinhardtii           AVCVIHANFVTADATSHWL.......LG.VQLIALYCVIAVAYFYR..............
Dictyostelium_discoideum            LLTI....CAAAASGIGYFVNKIKQQHG.LMLFAIFLLAGIIQYKYNINIDEGD.IPVID
Saccharomyces_cerevisiae            FIAVFLSNYLILDGESNWL.......EG.VMSLAMYILIAMAFFYY....PDEKTLDSIG
Caenorhabditis_elegans              FLAISLIFTLIAMFAQKYFLRR....IHSYSLVFIYISFFVFIGLSLDDILVWN......
Arabidopsis_thaliana                VGLLW...ALVIMPKKKMRLDKL...VG.GGLLAIYLCFLSLRLARVFGVLDTDR.....
Drosophila_melanogaster             VVTAAV..LYLGLLARRFVMGK.T..VG.YLSIIFYALFLIIACTLE...ILLNKEKMCD
Danio_rerio                         LLSIIF..LFLAVHINGWKLDW.K..LG.LVCLACYILFATLSILYELGIIGNNPIRSCS
Xenopus_laevis                      LFSIMF..IFVAIHLNGWKLDK.K..LG.VICLFMYLVFVTLSILYELGIIGNTPMVLCG
Gallus_gallus                       LGSVALTGTVILLSPREPSVERIK..SQRYKNTHLYLSLPVETLLEE.GIITGDATPLHG
Mus_musculus                        NISILF..LFLAVHFNGWKLDR.K..LG.VVCLVLYLGLATLSVLYEIGIIGNNRIRGCG
Homo_sapiens                        LGSVAL..TVLGIHLNKWRLDR.K..LG.VYVLVLYAIFLCFSIMIEFNVFTFVNLPMCR
```

**Bordetella_pertussis_Tohama_I**	. . .
**Aeromonas_veronii**	. . .
**Bordetella_pertussis_Mn_resister**	. . .
**Bordetella_bronchiseptica**	. . .
**Escherichia_coli**	. . .
**Clostridium_perfringens**	. . .
**Methanocaldococcus_jannaschii**	. . .
**Chlamydomonas_reinhardtii**	. . .
**Dictyostelium_discoideum**	P I A
**Saccharomyces_cerevisiae**	N S L
**Caenorhabditis_elegans**	. . .
**Arabidopsis_thaliana**	. . .
**Drosophila_melanogaster**	I Q D
**Danio_rerio**	D . .
**Xenopus_laevis**	D . .
**Gallus_gallus**	. . .
**Mus_musculus**	V . .
**Homo_sapiens**	E D D
