# Supplementary material for: A Unique Reverse Adaptation Mechanism Assists Bordetella pertussis in Resistance to Both Scarcity and Toxicity of Manganese
Source: mBio. 2021 Oct 26;12(5):e01902-21. doi: 10.1128/mBio.01902-21 (PMC8546581; doi:10.1128/mBio.01902-21)
Supplement: TABLE S1 [file mbio.01902-21-st001.pdf]

Supplementary Table 1. Strains and plasmids used in the study

Strain or plasmid	Description	Reference
**Strains**		
Escherichia coli		
SM10(λ*pir*)	Maintenance and mobilization of pSS4245 vector	[1]
XL1 blue	Cloning strain	
Bordetella pertussis		
Tohama I	Wild-type strain	[2]
$BP3410_{mutGG}$	Mutant carrying two point mutations within the riboswitch	This study
Δ*BP3410*	Deletion mutant lacking the *BP3410* gene	This study
Mn resister	Spontaneous mutant lacking the duplication within the *BP3410* gene	This study
Mn $resister_{C}$	Mutant lacking the duplication within the *BP3410* gene due to site-directed mutagenesis	This study
wt (pBBR)	Wild-type strain bearing empty pBBR1MCS plasmid	This study
Δ*BP3410* (pBBR)	Δ*BP3410* strain bearing empty pBBR1MCS plasmid	This study
Mn $resister_{C}$ (pBBR)	Mn $resister_{C}$ strain bearing empty pBBR1MCS plasmid	This study
Δ*BP3410* (pBBR:: *BP3410*)	Δ*BP3410* strain bearing plasmid containing wild type allele of the *BP3410* gene	This study
**Plasmids**		
pSS4245	Conjugation vector for allelic exchange in *Bordetella pertussis*	[3]
pBBR1MCS	Broad spectrum vector for cloning in $Gram^{-}$ bacteria	[4]

1. Simon, R., U. Priefer, and A. Pühler, *A Broad Host Range Mobilization System for In Vivo Genetic Engineering: Transposon Mutagenesis in Gram Negative Bacteria.* Biotechnology, 1983. **1** p. 784–791.
2. Kasuga, T., et al., *Studies on Haemophilis pertussis. III. Some properties of each phase of H. pertussis.* Kitasato Arch Exp Med, 1954. **27**(3): p. 37-47.
3. Inatsuka, C.S., et al., *Pertactin is required for Bordetella species to resist neutrophil-mediated clearance.* Infect Immun, 2010. **78**(7): p. 2901-9.
4. Kovach, M.E., et al., *pBBR1MCS: a broad-host-range cloning vector.* Biotechniques, 1994. **16**(5): p. 800-2.
