# Supplementary material for: A Unique Reverse Adaptation Mechanism Assists Bordetella pertussis in Resistance to Both Scarcity and Toxicity of Manganese
Source: mBio. 2021 Oct 26;12(5):e01902-21. doi: 10.1128/mBio.01902-21 (PMC8546581; doi:10.1128/mBio.01902-21)
Supplement: TABLE S2 [file mbio.01902-21-st002.pdf]

Supplementary Table S2. Primers used in this study

Purpose of the primers	Sequence (5′->3′)*
Construction of $BP3410_{mutP}$ mutant (upstream region)	F:TGAATTCTGTTGTACGCCACCGATT<br>R:AGCTAGCGGCAATCTGCCTGGCC
Construction of $BP3410_{mutP}$ mutant (downstream region)	F:TGCTAGCCGCGCACTGTCATTG<br>R:TGAATTCGAACACGTGCGAAGCCA
Construction of $BP3410_{GG}$ mutant (upstream region)	F:TTTGAATTCCAGCTCATACTCGCGCATGCC<br>R:GGATAGAGATGGTTACT**TT**CCAATGACAGTGCGC
Construction of $BP3410_{GG}$ mutant (downstream region)	F:TTTGAATTCGATCGCGCGGCCGCAGCT<br>R:GCGCACTGTCATTGG**AA**AGTAACCATCTCTATCC
Construction of Δ*BP3410* mutant (upstream region)	F:ATAGAGCTCGCACCCGCTCGTCGAG<br>R:ATAGCTAGCCATGATGACGCGGCCGGA
Construction of Δ*BP3410* mutant (downstream region)	F:ATAGCTAGCTGAGCCGCATGACGCCG<br>R:ATAGAGCTCGTTCAGGCCACGGCCTC
PCR amplicon visualizing duplication loss	F:CGGAAATCATGAACGCCCTGATC<br>R:AGCGTAGAAATGCCAGCCCA
RT-qPCR analysis of *BP3410* expression	F:CAAGGTCTTCGCAATCCAGG<br>R:CGTGTTCGTGTTCAAGGTGG
RT-qPCR analysis of *rpoB* expression	F:GCTGGGACCCGAGGAAAT<br>R:CGCCAATGTAGACGATGCC

*F, forward primer; R, reverse primer; restriction sites are underlined; nucleotides introducing mutations are in bold
